# Incidental DWI-Positive Lesions in 2 Cohorts of CAA and CADASIL: Prevalence, Distribution, and Associations With Clinical Variables

**DOI:** 10.1212/WNL.0000000000218160

**Published:** 2026-06-26

**Authors:** Annemieke ter Telgte, Anna M. de Kort, Anna Dewenter, Anna Kopczak, Benno Gesierich, H. Bea Kuiperij, Catharina J.M. Klijn, Marco Duering, Marcel M. Verbeek, Floris H.B.M. Schreuder

**Affiliations:** 1 VASCage - Centre on Clinical Stroke Research, Innsbruck, Austria;; 2 Department of Neurology, Medical University of Innsbruck, Austria;; 3 Department of Neurology, Donders Institute for Brain, Cognition and Behaviour, Radboud University Medical Center, Nijmegen, the Netherlands;; 4 Institute for Stroke and Dementia Research (ISD), LMU University Hospital, LMU Munich, Germany;; 5 Antaros Medical AB, Mölndal, Sweden;; 6 Translational Imaging in Neurology (ThINk), Department of Biomedical Engineering, Faculty of Medicine, University Hospital Basel and University of Basel, Switzerland; and; 7 Department of Human Genetics, Radboud University Medical Center, Nijmegen, the Netherlands.

## Abstract

**Background and Objectives:**

Incidental hyperintense lesions on diffusion-weighted imaging (DWI) are suggested as emerging marker of cerebral small vessel disease (SVD). To further determine their role in SVD, we aimed to describe their prevalence on high-resolution DWI in 2 distinct SVD types. Second, in each SVD type, we aimed to assess incidental DWI-positive lesion distribution and associations with clinical variables.

**Methods:**

Data from 2 hospital-based prospective cohorts in the Netherlands and Germany were used, which included patients meeting the modified Boston criteria for probable cerebral amyloid angiopathy (CAA, BIONIC study) and patients with a confirmed diagnosis of cerebral autosomal dominant arteriopathy with subcortical infarcts and leukoencephalopathy (CADASIL, VASCAMY study). In case of a stroke history, patients ≤3 months poststroke were excluded. 3T high-resolution baseline MRIs of patients with probable CAA and baseline, 18-month, and 36-month MRIs of patients with CADASIL were included. All MRI markers were rated following STRIVE-2. Within patient groups, we explored in univariable analyses the association between cardiovascular risk factors and MRI markers and incidental DWI-positive lesions and in multiple regression the association between fluid biomarkers and incidental DWI-positive lesions.

**Results:**

Baseline data were available for 43 CAA patients (mean baseline age 71 ± 6 years, 44% female) and 75 CADASIL patients (mean baseline age 53 ± 9.9 years, 62% female). Cross-sectionally, incidental DWI-positive lesions were detected in 24/43 (56% [95% CI 41%–70%]) CAA patients and 16/75 (21% [95% CI 14%–32%]) CADASIL patients. In CAA, 65% of lesions were located in the cortex, whereas in CADASIL, 95% of lesions were located in the subcortical white or gray matter. In CAA patients, DWI-positive lesions were significantly associated with increased neurofilament light chain (NfL) in serum and CSF, but not with other CSF, MRI, or cardiovascular risk factors. In CADASIL patients, DWI-positive lesions were significantly associated with increased serum NfL, increased white matter hyperintensity volume and lacune presence.

**Discussion:**

In CAA and CADASIL, the prevalence of incidental DWI-positive lesions is high, and lesions have disease-specific distribution, and associations with serum, CSF, and MRI biomarkers, suggesting that incidental DWI-positive lesions are a feature of SVD. Future studies should investigate their prognostic value.

## Introduction

Cerebral small vessel disease (SVD) is a disease of the smallest blood vessels in the brain and associated with increased risk of dementia and stroke.^1^ Features of SVD are commonly seen in elderly individuals on MRI), including white matter hyperintensities (WMH), lacunes, and cerebral microbleeds. As a potential mechanism underlying these MRI markers, diffusion-weighted imaging (DWI)–positive lesions are increasingly studied.^2^ Such lesions are small (≤20 mm on axial plane on DWI), suggestive of acute brain infarcts,^3^ and typically clinically overlooked due to their transient detectability and asymptomatic nature. However, incidental DWI-positive lesions are not a rare phenomenon, but much more common than symptomatic recurrent stroke.^4,5^

Using high-frequent serial MRI, it has been shown that incidental DWI-positive lesions are associated with progression of SVD MRI markers, and in part explain the origin of these markers.^2,6^ Consequently, the Standards for Reporting Vascular Changes on Neuroimaging 2 (STRIVE-2) proposed incidental DWI-positive lesions as emerging marker of SVD.^1^ However, further understanding of their characteristics and potential causes is needed before they can become an established marker of SVD.^1^ This may be achieved by studying incidental DWI-positive lesions in patients with well-defined SVD, preferably in the absence of concomitant causes of acute ischemia.^7^

Cerebral amyloid angiopathy (CAA) and cerebral autosomal dominant arteriopathy with subcortical infarcts and leukoencephalopathy (CADASIL) reflect 2 well-defined SVDs, each with specific diagnostic criteria allowing for an in vivo (probability) diagnosis, including genetic testing for CADASIL and the Boston criteria for CAA.^8,9^ CAA is marked by the deposition of amyloid beta (Aβ) in the walls of the pial arteries, cortical perforators and capillaries, and a primary cause of spontaneous lobar intracerebral hemorrhage (ICH).^8,10^ CAA is also associated with cortical cerebral microinfarcts, which can nowadays be detected in vivo with high-resolution MRI.^10-12^ CADASIL is the most common hereditary form of SVD affecting predominantly the perforating arterioles supplying the white and subcortical gray matter.^9^ Most CADASIL patients suffer from recurrent subcortical infarcts, with MRI being marked by severe WMH, lacunes, and microbleeds.^9^ Clinical manifestations of CADASIL occur usually at a younger age than in CAA, when other age-related comorbidities are often absent. As such, CADASIL may regarded as a disease model for pure SVD.^9,13^

Whereas CAA and CADASIL are each associated with a distinct intrinsic vessel pathology, in both diseases, vessel changes increase the risk for ischemia, which can be assumed as the shared final pathway.^10,14^ However, our understanding of the frequency and relevance of incidental DWI-positive lesions in SVD is limited as few studies have investigated incidental DWI-positive lesions on high-resolution DWI scans. Therefore, we aimed to describe the prevalence of incidental DWI-positive lesions in 2 CAA and CADASIL research cohorts, using high-resolution DWI. Secondly, in each SVD type we aimed to assess lesion distribution, and associations with clinical variables.

## Methods

### Participants

Patients with CAA were recruited into BIOmarkers for cogNitive Impairment due to Cerebral amyloid angiopathy (BIONIC),^15^ an observational study on CAA conducted between 2018 and 2023 at the Department of Neurology, Radboudumc, Nijmegen, the Netherlands.^16^ Study eligibility criteria included a diagnosis of probable CAA according to the modified Boston criteria,^17^ and no contraindications for 3T MRI and lumbar puncture. Patients with a history of symptomatic ICH needed to be >3 months post-ICH to exclude potential effects of the ICH or subsequent treatment on biomarker analyses. To determine disease-specific elevation of DWI lesion prevalence, we compared CAA patients with age-matched and sex-matched controls free of stroke and dementia. Controls were recruited parallel to BIONIC following the same procedures (CAFE study).^15^

Patients with CADASIL were recruited into Vascular and Amyloid predictors of neurodegeneration and cognitive decline in nondemented subjects (VASCAMY), an observational study conducted between 2015 and 2019 at the Institute for Stroke and Dementia Research, LMU University Hospital, Munich, Germany.^18^ Here, we focused on the CADASIL arm of VASCAMY. Patients underwent 3T MRI and standardized neuropsychological testing at baseline, and 18 and 36 months after baseline. Inclusion criteria were a diagnosis of CADASIL (cysteine-altering NOTCH3 mutation or skin biopsy) and age older than 18 years. Exclusion criteria were other major neurologic or psychiatric disease, diabetes mellitus with antidiabetic treatment (to rule out effects of long-term diabetes on brain structure and function), substance abuse, and MRI contraindications. Eighteen patients concurrently participated in other studies (15 participated in Zoom@SVDs, 2 in INVESTIGATE-SVDs, and 1 in Zoom@SVDs and INVESTIGATE-SVDs). None participated concurrently in the investigational trial TREAT-SVDs. For this study, we further excluded patients with a clinical diagnosis of stroke ≤3 months before the baseline DWI scan and patients with presence of other causes of acute ischemia than SVD, including known atrial fibrillation.

### MRI Acquisition

MRI data of CAA patients (and controls) were collected on a single 3T MRI scanner (PrismaFit, Siemens Healthineers, Erlangen, Germany), with a 32-channel coil. MRI sequences included: multishell DWI (1.7 mm isotropic voxels), and high-resolution 3D T1-weighted, T2, fluid-attenuated inversion recovery (FLAIR),^19^ and multiecho gradient echo T2*-weighted imaging to generate susceptibility-weighted imaging (SWI) scans.^15,20^

MRI data of CADASIL patients were collected with a 3T MAGNETOM Verio or Skyra scanner using a 32 or 64-channel head(-neck) coil (Siemens, Healthineers, Erlangen, Germany), respectively. Different protocols were used due to decommissioning of the Verio scanner in the longitudinal course of the study. Diffusion data (2.0-mm isotropic voxels) were collected using a single-shell (Verio, N = 66 images at baseline) or multishell protocol (Skyra, N = 9 images at baseline, and N = 70 images at follow-up). The acquisition parameters for all sequences have been described elsewhere.^18,21,22^

### DWI Data Processing

Diffusion data obtained with a multishell protocol were preprocessed, as described previously.^23^ Preprocessing of the single-shell diffusion data entailed denoising and removal of Gibbs artifact using tools from MRtrix (v3.0), and correction for susceptibility-induced distortions, motion and eddy currents using “eddy” and “topup” within the Functional Magnetic Resonance Imaging of the Brain Software Library (FSL; v5.0).^24,25^ We generated the DWI trace images separately for the different shells based on the arithmetic mean across diffusion-weighted directions and the mean diffusivity map (MD) for the b = 1,000 shell using “dtifit” from FSL.

### Assessment of SVD MRI Markers

The STRIVE-2 criteria for incidental DWI-positive lesions were applied.^1^ One rater with >7 years of experience (A.t.T.) manually screened all DWI trace images of all individuals using FSLeyes (version 1.6.1), unaware of other SVD MRI marker ratings, and considering both diffusion shells when available.^2^ All detected DWI hyperintensities were discussed with a second independent expert rater (M.D.) considering for each possible lesion, in addition to the diffusion trace images, the MD, T1, FLAIR, and SWI scan to rule out as much as possible artifacts and distinguish older lesions from incidental lesions. All DWI scans were collected in the context of research and not because of a clinical indication. Hence, none of the lesions fulfilled the criteria for recent small subcortical infarcts.^1^

Since hemorrhagic lesions can give rise to DWI distortions mimicking DWI-positive lesions,^26^ we decided on the following criteria: (1) we excluded DWI hyperintensities *within* lobar hematomas; (2) we identified a typical DWI artefact *surrounding* (small) hemorrhages and excluded these from our rating; and (3) we included punctate DWI hyperintensities indicative for a DWI-positive lesion located adjacent or in close proximity to hematoma but noted these as separate category (eFigure 1–2). We did not exclude DWI hyperintensities occurring close to cortical superficial siderosis (cSS) or cerebral microbleeds, since both lesions may in part have an ischemic origin.^2,27-29^

One rater (A.t.T.) manually drew masks of incidental DWI-positive lesions in FSLeyes.^2^ For each incidental DWI-positive lesion, we determined its volume in native space (mL), location using the T1 and FLAIR (cortex, white matter, subcortical gray matter, brainstem, cerebellar, or cortical-subcortical junction), and whether lesions fulfilled the size criteria for recent cortical microinfarct (i.e., ≤ 5 mm).^1,30^ To generate DWI lesion distribution maps, the DWI trace images were registered to the T1, and normalized nonlinearly to MNI space using Advanced Normalization Tools. The combined transformations were then applied to the DWI lesion masks.

Conventional MRI markers were defined according to STRIVE and were rated by other raters.^1^ For BIONIC and CAFE, WMH volumes, including WMH in the brainstem and subcortical gray matter regions, were segmented with an in-house developed deep learning model.^31^ All WMH segmentations were visually checked, and no major errors were observed. To exclude gliosis surrounding large hematoma from the WMH segmentations, we manually drew masks of the hematoma guided by the FLAIR scan. In addition, we drew masks of larger ischemic infarcts not due to SVD to exclude these from the WMH volume estimation. Intracranial volume was used for normalization and calculated segmentation-based using FreeSurfer SAMSEG. For CAA, all other MRI markers were rated by AdK, supervised by FHBMS. These included presence of lacunes, including within the cerebellum or brainstem based on FLAIR and T1 scans, presence and number of cerebral microbleeds (1: 2–4 microbleeds, 2: ≥5 microbleeds), presence of cSS (1: focal, 2: disseminated), low (≤20) or high (≥21) number of perivascular spaces in the centrum semiovale, and Fazekas score for WMH.^15^ Based on the latter 4 markers, the CAA-SVD summary score was calculated ranging from 0-6, reflecting CAA severity.^15,32^ In addition, microbleeds number was categorized as 1: 1–2 microbleeds, 2: 3–5 microbleeds, 3: 6–10 microbleeds, and 4: >10 microbleeds. For VASCAMY, derivation of brain volumes and MRI markers of SVD has been outlined before.^21,22^

Finally, using single-shell diffusion MRI data, we determined the peak width of skeletonized MD (PSMD, release 1.8.3), a fully automated and quantitative MRI marker of SVD derived from diffusion tensor imaging.^21^ PSMD can be automatically executed using an open-access tool. In CAA patients, regions of large hematoma or large ischemic infarcts were excluded before calculating PSMD.

### Clinical Variables

Age, sex, and educational level were given through self-report. Hypertension was defined as use of antihypertensive medication or an on-site mean blood pressure >140/90 mm Hg. In BIONIC, diabetes was defined as presence of diabetes type 1 or 2 in medical history, and hypercholesterolemia as use of a lipid-lowering drug or total cholesterol >6.2 mmol/L.^15^ In VASCAMY, diabetes and hypercholesterolemia were defined by the use of an antidiabetic or lipid-lowering medication, respectively. In both studies, the history of stroke (ischemic or hemorrhagic) was based on careful review of each patient's medical history.

As marker of neuroaxonal damage known to be increased in CAA and CADASIL,^33-35^ we assessed serum neurofilament light chain (NfL) (CAA: ELLA automated immunoassay system, Biotechne, Minneapolis; CADASIL: Simoa HD-1, Quanterix, Lexington), and CSF NfL in CAA patients (ELLA automated immunoassay system, Biotechne, Minneapolis). Furthermore, we explored in CAA patients associations between incidental DWI-positive lesions and CSF Aβ40, Aβ42, phosphorylated tau, and total tau (Lumipulse chemiluminescent assay, Fujirebio, Ghent, Belgium). Procedures for fluid biomarker analysis have been described previously.^15,16,33,34^ For both studies, fluid biomarkers were collected at baseline.

### Statistical Analysis

Data are reported as median [interquartile range, IQR] or proportions (%). Normality of the data was tested using the Shapiro-Wilk test. The significance level *α* was set at 0.05 for all exploratory analyses. We assessed the difference in prevalence of incidental DWI-positive lesions in CAA vs controls using the Fisher exact test and the difference in incidental DWI-positive lesion volume between patients with CAA vs CADASIL using the Mann-Whitney *U* test. In univariable analyses, we assessed within patients with CAA and CADASIL separately the association between baseline risk factors and presence of incidental DWI-positive lesions using the Mann-Whitney *U* test and chi-squared or Fisher exact test where applicable. For patients with CAA, in sensitivity analyses we excluded patients with causes of acute ischemic stroke other than SVD, including a medical history of atrial fibrillation or clinical or imaging evidence of non-SVD ischemic stroke. As the CADASIL patients were included in a longitudinal study, the presence of incidental DWI-positive lesions in these patients was defined as having at least one incidental DWI-positive lesion at any timepoint. In a sensitivity analysis, we repeated the analysis including only baseline data.

The association between baseline presence of incidental DWI-positive lesions and baseline fluid biomarkers was assessed using multiple linear regression models, including one fluid biomarker as dependent variable and presence of incidental DWI-positive lesions, age, sex, and additionally any covariate with *p* < 0.05 in univariable analyses as independent variables, respectively. Fluid biomarker levels were log-transformed due to non-normality of residuals in most instances. In case of a significant result for DWI-positive lesions, we explored Spearman correlation between the fluid biomarker level and the number of incidental DWI-positive lesions. Statistical analyses were performed in R. Missing data were not imputed.

### Standard Protocol Approvals, Registrations, and Patient Consents

All participants provided written informed consent. The medical ethics committee Arnhem-Nijmegen approved BIONIC and CAFE. VASCAMY was approved by the ethics committee of LMU Munich.

### Data Availability

Anonymized data not published within this article will be made available by reasonable request from any qualified investigator.

## Results

### Cohort Description

**Figure 1:**
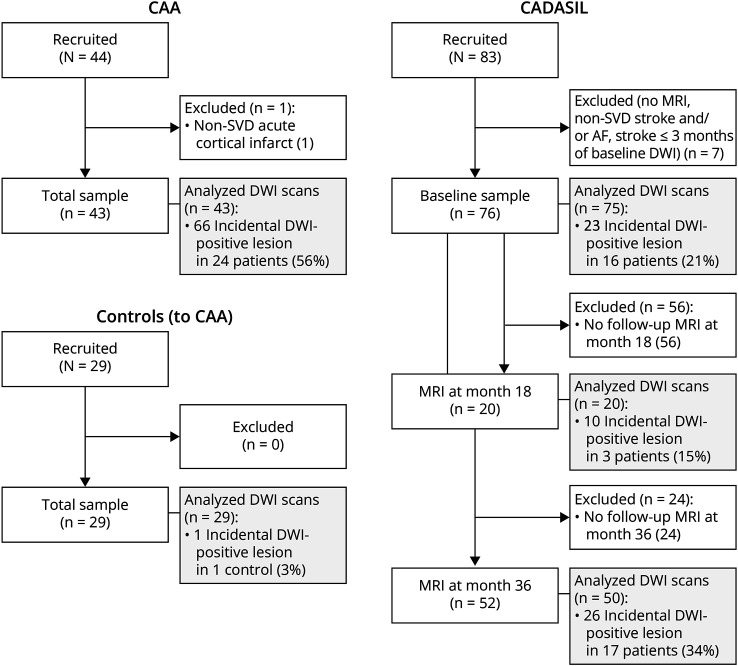
Flowchart of Participants Included in the Current Study AF = atrial fibrillation; CAA = cerebral amyloid angiopathy; CADASIL = cerebral autosomal dominant arteriopathy with subcortical infarcts and leukoencephalopathy; DWI = diffusion-weighted imaging; SVD = small vessel disease.

In total, 44 patients with probable CAA and 29 controls were recruited. One CAA patient was excluded after detecting a large cortical DWI-positive lesion presumably unrelated to SVD (eFigure 3), resulting in a sample of N = 43 patients (Figure 1). Patients with CAA were on average 71 (±6) years, and 19/43 (44%) were female.

In total, 83 patients with CADASIL were recruited. Two did not have any MRI and 5 did not fulfil inclusion criteria for the current study, resulting in a total sample of 76 patients. At baseline, one patient did not have MRI data available. Of the total sample, 20 underwent MRI at 18 months, and 52 at 36 months. Importantly, during the course of the VASCAMY study, the 18-month follow-up visit was removed to reduce the travel burden for patients leading to a lower sample size at month 18. For 2 patients with a stroke ≤3 months of a follow-up DWI scan, we excluded the follow-up DWI scan. Overall, 145 DWI scans were available for assessment of incidental DWI-positive lesions. The mean baseline age of the total CADASIL sample was 53 (±9.9) years and 47/76 (62%) were female.

### Presence and Characteristics of Incidental DWI-Positive Lesions

In CAA, 66 incidental DWI-positive lesions were detected in 24/43 patients (56%, [95% CI 40%–71%]). Of these 24 patients, 9 had one incidental DWI-positive lesion, 7 had 2 lesions, and 8 had ≥4 lesions with a maximum of 10 lesions detected in one patient. The prevalence of incidental DWI-positive lesions in CAA was significantly increased compared with controls (1 control had 1 incidental DWI-positive lesion, 3% [95% CI 0.01%–18%], *p* < 0.001).

In CADASIL, at baseline 23 incidental DWI-positive lesions were detected in 16/75 patients (21%, [95% CI 14%–32%]). The prevalence of DWI lesions at months 18 and 36 were 3/20 (15%, [95% CI 5%–36%]) and 17/50 (34%, [95% CI 22%–48%]), respectively. Across all time points, 59 incidental DWI-positive lesions were detected in 27/76 patients (36%, [95% CI 26%–47%]). Of these 27 patients, 19 had a single or multiple incidental DWI-positive lesion(s) at one time point, and 8 had incidental DWI-positive lesions at multiple time points. Considering the number of incidental DWI-positive lesions per MRI scan, 24 DWI scans contained a single lesion, 6 DWI scans contained 2 lesions, 1 DWI scan contained 3 lesions, and 5 DWI scans contained 4 lesions.

**Figure 2:**
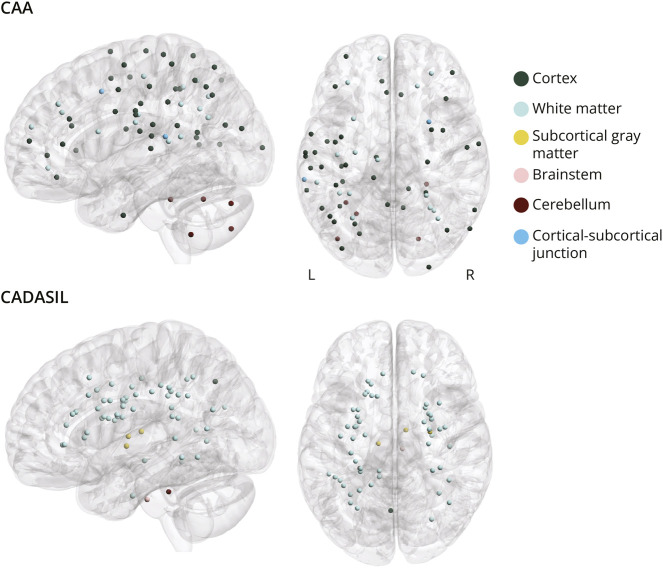
3D Distribution Map of Incidental DWI-Positive Lesions in CAA and CADASIL The distribution of incidental DWI-positive lesions was displayed using BrainNet Viewer, using a T1-weighted MNI-152 template as background image (Surf Ice tool). CAA = cerebral amyloid angiopathy; CADASIL = cerebral autosomal dominant arteriopathy with subcortical infarcts and leukoencephalopathy; DWI = diffusion-weighted imaging; MNI = Montreal neurological institute.

**Figure 3:**
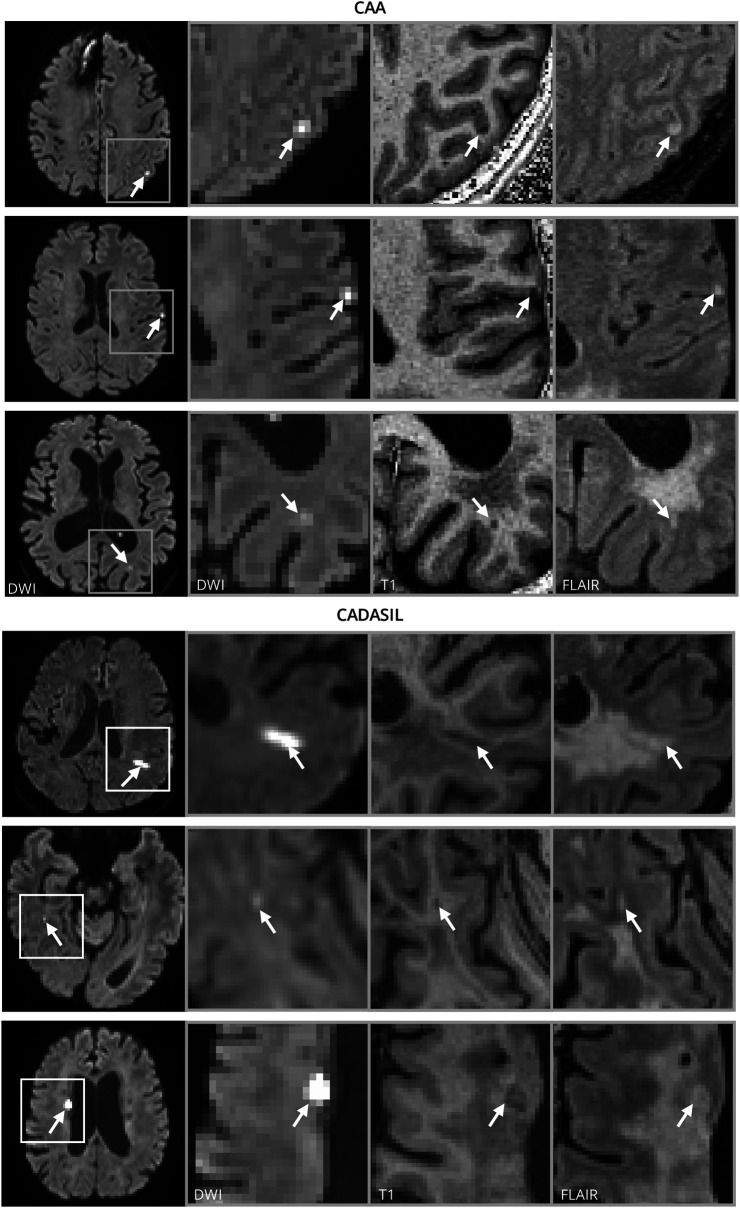
Examples of Incidental DWI-Positive Lesions in CAA and CADASIL on DWI (b = 1,000 s/mm^2^ trace), T1 and FLAIR CAA: shown are 2 cortical (top and middle row) and one white matter incidental DWI-positive lesions (bottom row). CADASIL: shown are 3 white matter incidental DWI-positive lesions. CAA = cerebral amyloid angiopathy; CADASIL = cerebral autosomal dominant arteriopathy with subcortical infarcts and leukoencephalopathy; DWI = diffusion-weighted imaging; FLAIR = fluid-attenuated inversion recovery.

Incidental DWI-positive lesion characteristics differed between CAA and CADASIL. In CAA patients, the median volume of incidental DWI-positive lesions was significantly smaller compared with CADASIL patients (0.015 mL [IQR 0.010–0.023] vs 0.024 mL [IQR 0.016–0.084], *p* = 0.001). In CAA, 43 (65%) incidental DWI-positive lesions were in the cerebral cortex and met the size criteria for recent cortical microinfarct, 16 were in the white matter, 2 were at the cortical-subcortical junction, and 5 were in the cerebellum. Of the 16 white matter lesions, 14 (88%) were lobar, that is, located near the cortex (Figures 2 and 3). Three incidental DWI-positive lesions were close to lobar ICH (eFigure 2). Of the 15 CAA patients with multiple incidental DWI-positive lesions, 7 patients had lesions in different brain locations and 8 patients in a single brain location.

In CADASIL patients, most, that is, 53/59 (90%), incidental DWI-positive lesions were in the white matter, 3 were in the subcortical gray matter, 1 in the cerebral cortex (corresponding to a recent cortical microinfarct, eFigure 4), 1 in the cerebellum, and 1 in the brainstem.

### Associations With Clinical Variables

Table 1 presents baseline characteristics for patients with and without any incidental DWI-positive lesion for CAA and CADASIL separately. In CAA, the presence of incidental DWI-positive lesions was not significantly associated with demographics or cardiovascular risk factors. Similarly, whereas most SVD MRI markers were increased in CAA patients with incidental DWI-positive lesions, none of these differences were significant. In sensitivity analyses excluding 8 patients with possible other causes for DWI-positive lesions (2 patients with 2 incidental DWI-positive lesions, and 6 without), results remained stable except for a significant difference in the categorized number of microbleeds (DWI-positive: 3.5 [2–4]; DWI-negative: 2 [2–3]; *p* = 0.009) and PSMD (DWI-positive: 3.7 (1.1) 10^−4^ mm^2^/s; DWI-negative: 2.9 (0.1) 10^−4^ mm^2^/s; *p* = 0.018).

**Table 1:** Baseline Characteristics of Patients With and Without Any Incidental DWI-Positive Lesion in CAA and CADASIL

	CAA			CADASIL		
	DWI-positive lesion n = 24	No DWI-positive lesion n = 19	*p* Value	Any DWI-positive n = 27	No DWI-positive lesion n = 49	*p* Value
Demographics						
Age, y, median [IQR]	71 [67–74]	73 [65–77]	0.816	55 [50–59]	53 [44–60]	0.591
Female sex, n (%)	10 (42)	9 (47)	0.764	19 (70)	28 (57)	0.256
Educational level, median [IQR]^a^	5 [5–6]	6 [5–6]	0.949	13 [12–15]	13 [13–16]	0.471
Cardiovascular risk factors and medical history						
Hypertension, n (%)	15 (63)	11 (58)	1.00	9 (33)	7 (14)	0.051
Diabetes, n (%)	1 (4)	2 (11)	0.575	2 (7)	2 (4)	0.612
Hypercholesterolemia, n (%)	15 (68)	11 (58)	0.533^b^	12 (44)	25 (51)	0.583
Smoking, ever, n (%)	15 (63)	11 (58)	1.00	18 (67)	28 (57)	0.416
History of stroke, n (%)^c^	5 (21)	6 (32)	0.495	13 (48)	26 (53)	0.682
Imaging markers						
WMH volume, % of ICV, median [IQR]	1 [0.3–1.6]	0.6 [0.3–1.1]	0.231	7 [4–8]	4 [3–7]	0.036
Lacune, presence, n (%)	5 (21)	2 (11)	0.437	24 (89)	26 (53)	0.002
Microbleed, presence, n (%)	22 (92)	18 (95)	1.00	13 (48)	21 (43)	0.657
Microbleed, number, median [IQR]^d^	4 [2–4]	2 [2–3]	0.147	0 [0–2]	0 [0–1]	0.633
Cortical superficial siderosis distribution, median [IQR]^e^	2 [0–2]	1 [0–2]	0.368	—	—	—
CAA-SVD score, median [IQR]^f^	4.5 [4–6]	4 [3–5]	0.716	—	—	—
PSMD, 10^−4^ mm^2^/s, median [IQR]	3.5 [2.9–4.3]	3.0 [2.6–3.3]	0.092	6.1 [4.7–7.2]	4.9 [3.8–6.4]	0.080^g^

Abbreviations: CAA = cerebral amyloid angiopathy; CADASIL = cerebral autosomal dominant arteriopathy with subcortical infarcts and leukoencephalopathy; DWI = diffusion-weighted imaging; ICV = intracranial volume; IQR = interquartile range; PSMD = peak width of skeletonized mean diffusivity; SVD = small vessel disease; WMH = white matter hyperintensities.

a In patients with CAA, educational level was determined according to the 7-point Verhage^36^ scale. In patients with CADASIL educational level was expressed as years of education.

b Data is missing for 2 patients.

c For VASCAMY history of stroke also included transient ischemic attack (TIA).

d The number of microbleeds was grouped as follows: 0 = 0 microbleeds, 1 = 1–2 microbleeds, 2 = 3–5 microbleeds, 3 = 6–10 microbleeds, 4 = > 10 microbleeds.

e Cortical superficial siderosis was classified as follows: 0 = none, 1 = focal, 2 = disseminated.

f The CAA-SVD summary score ranged from 0-6.

g Data is missing for 9 patients.

In CADASIL patients, the presence of incidental DWI-positive lesions was significantly associated with WMH volume and presence of lacunes (all *p*-values <0.05), with a trend for hypertension. In sensitivity analysis focusing on presence of incidental DWI-positive lesions at baseline only, results remained stable, except for a nonsignificant association for presence of lacunes (*p* = 0.071).

Serum and CSF biomarkers were available for >85% of the CAA patients, and serum NfL was available for 69% of CADASIL patients. The main reason for missing CSF data was failure of lumbar puncture and for missing NfL data timing of the analyses.

**Figure 4:**
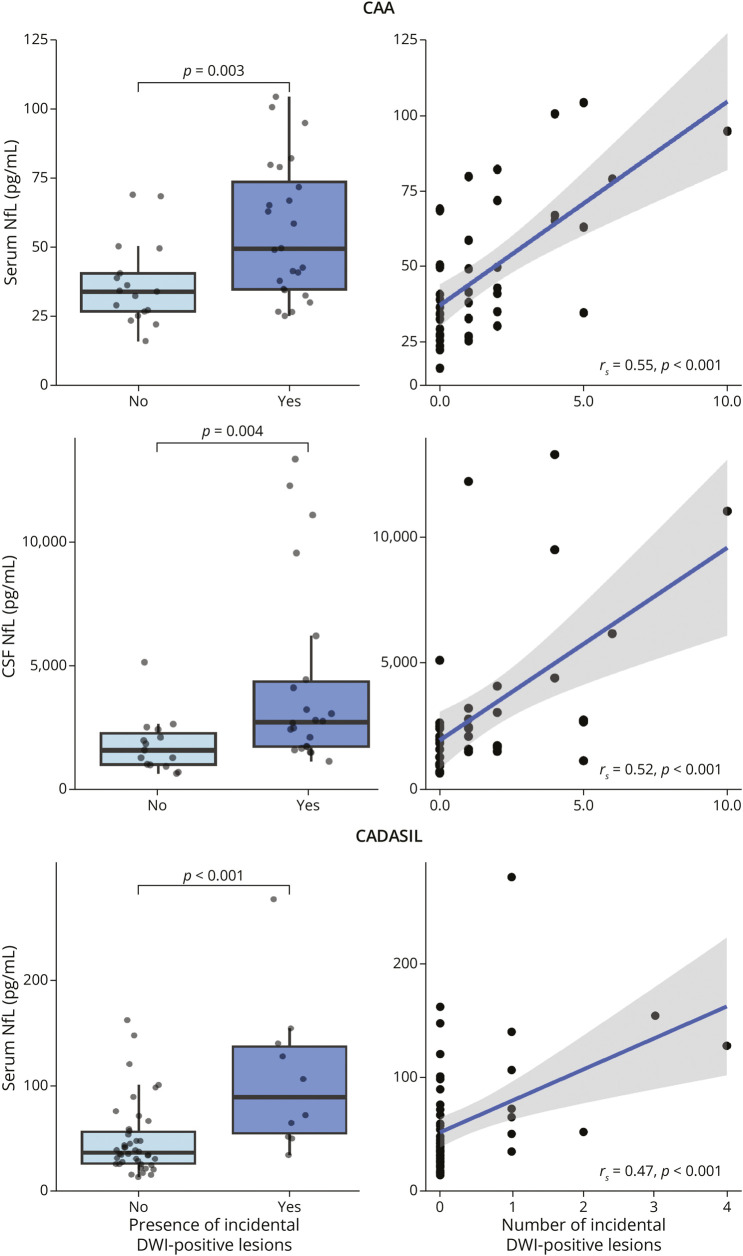
Association Between Serum and CSF NfL and the Presence of Incidental DWI-Positive Lesions The dots represent the individual participant-level data. The *p* values for the box plots represent *p* values obtained in multiple linear regression adjusted for age and sex, as presented in Table 2. Fluid biomarkers were collected at baseline. DWI = diffusion-weighted imaging; NfL = neurofilament light chain.

In CAA, incidental DWI-positive lesions were significantly associated with increased serum and CSF NfL (Figure 4 and Table 2). The number of incidental DWI-positive lesions and NfL levels were positively correlated (serum: *r*_*s*_ = 0.55, *p* < 0.001; CSF: *r*_*s*_ = 0.52, *p* = 0.001). In sensitivity analyses results did not change. The remaining CSF biomarkers (i.e., Aβ40, Aβ42, phosphorylated tau and total tau) were not significantly associated with the presence of incidental DWI-positive lesions.

**Table 2:** Association Between the Presence of Incidental DWI-Positive Lesions and Log-Transformed Fluid Biomarker Levels

	R^2^	B (SE B)	95% CI	*p* Value
CAA				
Outcome				
Serum NfL	0.21	0.182 (0.057)	0.066 to 0.298	0.003
CSF NfL	0.19	0.31 (0.099)	0.108 to 0.510	0.004
CSF Aβ40	−0.02	−0.036 (0.039)	−0.114 to 0.043	0.362
CSF Aβ42	−0.07	−0.029 (0.057)	−0.144 to 0.086	0.922
CSF Phosphorylated tau	−0.05	0.029 (0.076)	−0.125 to 0.183	0.702
CSF Total tau	−0.01	0.072 (0.070)	−0.070 to 0.214	0.311
CADASIL				
Outcome				
Serum NfL	0.51	0.739 (0.169)	0.400 to 1.078	< 0.001

Abbreviations: CAA = cerebral amyloid angiopathy; CADASIL = cerebral autosomal dominant arteriopathy with subcortical infarcts and leukoencephalopathy; DWI = diffusion-weighted imaging; NfL = neurofilament light chain.

Models were adjusted for age and sex. B = unstandardized beta; *p* value reflects the significance value for the predictor presence of incidental DWI-positive lesion.

In CADASIL, incidental DWI-positive lesions were significantly associated with increased serum NfL, also after further adjustment for WMH volume and presence of lacunes. A positive association between number of incidental DWI-positive lesions and NfL levels was observed (*r*_*s*_ = 0.45, *p* <0 .001).

## Discussion

We demonstrated a point prevalence of incidental DWI-positive lesions of 56% in probable CAA and 21% in CADASIL. In CAA, most incidental DWI-positive lesions were small and located in or near the cortex. In CADASIL, incidental DWI-positive lesions were larger and mostly in the white and subcortical gray matter. In both CAA and CADASIL, serum NfL was significantly associated with presence and number of incidental DWI-positive lesions. In CAA patients, in whom additionally CSF was collected, we observed similar associations with CSF NfL. In CADASIL but not in CAA, incidental DWI-positive lesions were significantly associated with increased WMH volume and presence of lacunes.

The prevalence of incidental DWI-positive lesions in CAA and CADASIL has been previously studied, although mostly in the clinical context using clinical DWI scans, which typically have large voxel sizes. For CAA, data are mostly derived from patients suffering (sub)acute ICH.^27,37-39^ However, one longitudinal cohort including CAA patients with or without a history of acute ICH reported a point prevalence of 18%, with 35% of patients having incidental DWI-positive lesions at any time point.^40^ For CADASIL, previous studies observed a point prevalence of incidental DWI-positive lesions of 8.4% and 10.5% and of acute cerebral microinfarcts in 22% of patients.^41-43^ Our study extends prior findings by demonstrating a high prevalence of incidental DWI-positive lesions among patients with CAA and CADASIL when high-resolution DWI is applied, and provides an explanation for the high prevalence of chronic cortical cerebral microinfarcts observed in patients with CAA.^3,10,11^

Importantly, we demonstrated a disease-specific distribution of incidental DWI-positive lesions, largely matching the underlying SVD. Incidental DWI-positive lesions in CAA primarily corresponded to cortical cerebral microinfarcts, whereas DWI-positive lesions in CADASIL were located predominantly in the white and subcortical gray matter. The pathophysiology of incidental DWI-positive lesions is still unknown and may be heterogeneous. One postmortem study including patients with CAA performed serial sectioning to identify the culprit vessels causing cortical microinfarcts. This study demonstrated that both acute and chronic cortical cerebral microinfarcts were located in regions with increased numbers of CAA-positive vessels, and culprit vessels were characterized by a loss of smooth muscle cells and smaller vessel lumen, increasing the chance for inadequate blood flow regulation and consequently ischemia.^10^ It has been shown that subcortical microinfarcts are more strongly related to atherosclerosis and arteriolosclerosis compared with CAA.^44^ As these vascular pathologies can coexist in older patients with sporadic CAA, part of the subcortical incidental DWI-positive lesions observed in CAA may be related to a hypertension-related SVD. Conversely, our data suggest that cortical cerebral microinfarcts are a rarer phenomenon in CADASIL but can be detected occasionally on high-resolution in vivo imaging. These findings are supported by a recent in vivo study and 3 postmortem studies among a few patients with CADASIL, which observed cortical cerebral microinfarcts in these patients.^14,41,45,46^ One of these studies described the vessel changes in regions close to the microinfarcts, which comprised NOTCH3-positivity, particularly in the leptomeningeal arterioles, and again smaller vessel lumen.^14^ Altogether, although CAA and CADASIL are each associated with distinct SVDs, the disease-specific distribution of incidental DWI-positive lesions suggests that these lesions are a feature of SVD and a common final pathway of both SVD types.

In CADASIL, associations with WMH and lacunes and a trend for hypertension further support the hypothesis that incidental DWI-positive lesions are a feature of SVD. However, in CAA no such associations were observed. The absence of associations between incidental DWI-positive lesions and cardiovascular risk factors is in line with previous data^40^ and may suggest that incidental DWI-positive lesions in CAA are more driven by Aβ accumulation. On the other hand, in contrast to prior work, we did not observe an association between incidental DWI-positive lesions and cSS, a feature characteristic for CAA, although our data point towards increased SVD markers among CAA patients with incidental DWI-positive lesions. The observed findings therefore, need to be further investigated in larger data sets. The low sample size and the possibility for misclassification of patients as DWI-negative, resulting from the transient detectability of DWI-positive lesions, may have complicated statistical analyses.

Incidental DWI-positive lesions were associated with increased serum and CSF NfL. NfL is a marker for neuroaxonal damage, and increased after ischemic stroke and in patients with recurrent incident infarcts.^34,47^ Previous studies demonstrated that NfL is also increased in CAA and CADASIL compared with controls and positively associated with SVD severity.^33-35^ Here we demonstrate within CAA and CADASIL patients specifically, elevated NfL levels among those with incidental DWI-positive lesions. It is unknown whether small DWI-positive lesions lead to measurable increases in NfL. However, supported by previous findings showing an association between serum NfL and both recent small subcortical infarcts and incident infarcts,^47,48^ and the observed positive association between NfL and number of incidental DWI-positive lesions, we suggest that incidental DWI-positive lesions may potentially affect NfL values.

Our results have clinical implications. First, while CAA is primarily associated with hemorrhages, recent insights indicate that nonhemorrhagic lesions, including WMH, may precede hemorrhagic complications.^49^ Our data underline a role for ischemic lesions in CAA, which may occur years before symptomatic ICH as incidental DWI-positive lesions were also detected in patients without a history of symptomatic ICH or cSS. Second, incidental DWI-positive lesions are commonly detected in patients suffering acute symptomatic ICH, both in patients with CAA or hypertension as underlying cause of ICH.^37-39^ It is unknown whether lesions are related to the acute ICH, subsequent therapy, or the underlying vasculopathy causing the ICH. However, the high prevalence of incidental DWI-positive lesions observed in CAA demonstrated here, together with data showing that the prevalence of incidental DWI-positive lesions remains high even in subacute ICH, suggest that incidental DWI-positive lesions detected in acute ICH patients may be part of the underlying vasculopathy.^39,50^ Third, the high prevalence of incidental DWI-positive lesions suggests that these lesions eventually may be detected in the majority of patients with CAA and CADASIL if repetitive imaging is applied, highlighting a mechanism for cognitive decline in patients with CAA and CADASIL. Because of the increased frequency of incidental DWI-positive lesions compared with symptomatic recurrent stroke as well as their acute nature, incidental DWI-positive lesions may be considered an additional imaging outcome for future clinical trials, or a marker to select patients with active disease who will likely progress.^4,5,38^ Finally, small cortical incidental DWI-positive lesions may be an additional nonhemorrhagic diagnostic marker for CAA along the existing Boston criteria, which should be further studied.^8^ Longitudinal studies on CAA, including the HIFI-CAA study (NCT06128824), will provide more insight into the frequency and prognostic value of incidental DWI-positive lesions in CAA patients.

Strengths of the study include its prospective study design, the inclusion of high-resolution DWI, optimized for the detection of incidental DWI-positive lesions, including microinfarcts, and the rating of all DWI scans by the same experienced rater. Nonetheless, the study also has limitations. First, because of the transient detectability of incidental DWI-positive lesions on MRI, misclassification of patients may have occurred. For patients classified as DWI-negative, lesions may simply have been missed leading to an underestimation of the prevalence of incidental DWI-positive lesions. As illustrated by the longitudinal data available for the patients with CADASIL, applying follow-up MRI increases the number of patients identified with a DWI-positive lesion and reduces the risk of misclassification. However, follow-up data were not available for patients with CAA. Furthermore, while we excluded patients with CADASIL with other vascular pathologies than SVD (e.g., patients with atrial fibrillation), for patients with CAA we ran sensitivity analyses excluding patients with known copathologies that could cause acute ischemia to not further reduce the sample size for CAA patients. In CAA patients, we did not systematically assess for cardio-embolic causes or carotid artery stenosis. Consequently, we cannot rule out that some incidental DWI-positive lesions had another cause than SVD. In VASCAMY, 2 different scanners and diffusion protocols were used. We have no data on how this may affect the detection of incidental DWI-positive lesions, which should be investigated further. Cardiovascular risk factor treatment may affect the frequency of incidental DWI-positive lesions. However, our sample sizes were too small to include these modifying variables in our analysis. Finally, as BIONIC and VASCAMY included an intensive day of measurements, both studies likely recruited less-disabled patients. This potential selection bias may limit the generalizability of current results to more severely affected patients who may have a higher prevalence of incidental DWI-positive lesions following our observations on associations between incidental DWI-positive lesions and SVD MRI markers. Moreover, in BIONIC, study participation rate was ∼50% since many eligible patients were unwilling to undergo lumbar puncture.

In conclusion, this study demonstrated that incidental DWI-positive lesions are common in CAA and CADASIL. The location and size of these lesions largely reflected the putative underlying SVD, suggesting that these lesions are a feature of SVD.

## Glossary

Aβ: amyloid beta
BIONIC: BIOmarkers for cogNitive Impairment due to Cerebral amyloid angiopathy
CAA: cerebral amyloid angiopathy
CADASIL: cerebral autosomal dominant arteriopathy with subcortical infarcts and leukoencephalopathy
cSS: cortical superficial siderosis
DWI: diffusion-weighted imaging
FLAIR: fluid-attenuated inversion recovery
ICH: intracerebral hemorrhage
IQR: interquartile range
NfL: neurofilament light chain
SVD: small vessel disease
SWI: susceptibility-weighted imaging
WMH: white matter hyperintensities
